# Undiagnosed chronic obstructive pulmonary disease in patients admitted to an acute assessment unit

**DOI:** 10.1080/20018525.2017.1292376

**Published:** 2017-03-08

**Authors:** Karin D. Eikhof, Kristine R. Olsen, N. C. H. Wrengler, Carl Nielsen, Uffe Bodtger, Ingrid L. Titlestad, Ulla M. Weinreich

**Affiliations:** ^a^ The Acute Assessment Unit, Aalborg University Hospital, Aalborg, Denmark; ^b^ Department of Respiratory Medicine, Naestved Hospital, Naestved, Denmark; ^c^ Department of Respiratory Medicine, Odense University Hospital, Odense, Denmark; ^d^ Department of Respiratory Diseases, Aalborg University Hospital, Aalborg, Denmark; ^e^ Institute for Regional Health Research, University of Southern Odense, Denmark; ^f^ Department of Respiratory Medicine, Zealand University Hospital, Roskilde, Odense, Denmark; ^g^ The Clinical Institute, Aalborg University Hospital, Aalborg, Denmark

**Keywords:** COPD, spirometry, acute illness, obstructive lung disease

## Abstract

**Introduction**: Chronic obstructive pulmonary disease (COPD) is very prevalent worldwide, yet underdiagnosed.

**Aim**: This study investigates feasibility of performing spirometry in patients in need of acute hospital admission as well as the prevalence of undiagnosed COPD in the same cohort.

**Methods**: During a two-week period, all patients admitted to three large acute assessment units were evaluated. Patients ≥ 18 years, able to perform spirometry, with no surgery to the thorax or abdomen within the last weeks and no known COPD was included. Patients with FEV1/FEV6 ≤ 0.7 or FEV1 < 80% or FEV6 < 80% were offered follow-up visit after 6 weeks.

**Results**: Of the 1145 admitted patients, 46% were eligible: 28% of those had an abnormal spirometry. The offered follow-up visit was attended by 51% and in this group 17% were diagnosed with lung disease. COPD was the most prevalent diagnosis (73%), and 2/3 was in GOLD group A. In total, 75% of the patients with airflow obstruction at the initial examination remained obstructive.

**Conclusion**: Performing spirometry in patients in need of acute hospital admission is feasible, abnormal findings are common, and COPD is the most prevalent diagnosis.

## Introduction

The prevalence of chronic obstructive pulmonary disease (COPD) is still increasing worldwide.[1] Early diagnosis of COPD is of great importance, as lung function level is associated with increased morbidity and mortality.[2,3] However, COPD is unfortunately also still underdiagnosed,[4] and patients have on average reduced lung function with 43% at time of diagnosis.[5] At the time of diagnosis patients with COPD have a history of a high degree of health care utilization and hospitalization and only have perceived symptoms in terms of mild dyspnea.[6–8]

Previous studies have identified undiagnosed COPD patients during hospital admissions.[9–12] However, none of these studies have included follow up on the patients identified in the screening process.

In Denmark about 640,000 people, equivalent to approximately 11% of the population, mean age 41 years old, are in need of acute hospital admission every year.[13,14] About 27% of the those are multi morbid.[13] Approximately 25% are discharged after 24 h admission, directly from the acute assessment unit (AAU) and within 36 h the majority have been transferred to specialized wards for further treatment.[13] Thus, we hypothesize that a proportion of the patients admitted to the AAU have undiagnosed COPD and that COPD can be diagnosed whilst patients are hospitalized due to acute illness.

The aim of this study is to investigate the feasibility of performing spirometry in patients admitted for acute assessment in hospital within 24 h of admission and to investigate the prevalence of COPD amongst these patients. Furthermore, to investigate whether a change in lung function could be detected when patients were re-examined 6 weeks after the admission.

## Material and methods

From 7 to 18 September 2015 all patients admitted to the AAUs in three Danish Hospitals were evaluated for this study. Patients 18 years of age and older, not previously diagnosed with COPD, able to understand Danish and to perform a spirometry were included in the study. Patients who had undergone thoracic surgery within 8 weeks, abdominal surgery within 4 weeks and had myocardial infarction within 4 weeks were excluded. Patients’ age, gender, height, weight smoking history and pack years were registered and their symptoms were investigated with both the Medical Research Council (MRC; 1–5) score and the COPD Assessment Test (CAT). Furthermore, patients were asked if they previously had undergone spirometry. Spirometry was performed by trained personnel, who all had undergone specific training in performing spirometry, according to American Thoracic Society/European Respiratory Society Task Force. Patients were sitting in an upright position, using Vitalograph COPD 6 (Vitalograph©, Buckingham, UK) registering forced expiratory volume in the first second (FEV1) and in the sixth second (FEV6) in liters as well as in % of expected value, using European reference values.[15] The ratio between FEV1/FEV6 was registered. Patients with a FEV1 or FEV6 below 80% of expected value or FEV1/FEV6 < 0.7 were offered a follow up in the Hospital Outpatient Clinic 6 weeks after the hospitalization. At this visit, patients were seen by a pulmonologist and both a pre- and post-bronchodilator spirometry was performed, using the spirometer available in the individual hospitals (Spida spirometry software Version 5, MicroMedical, CareFusion Health, San Diego, CA, USA; Odense; Næstved). FEV1 in liters and in % of expected value was registered as well as forced vital capacity (FVC) in both liters and % of expected value and the ratio between FEV1 and FVC. Furthermore, patients’ dyspnea was re-assessed using MRC score and patients were asked to state number of chest infections in the preceding year. Based on this, patients were stratified according to the 2016 Global Initiative for Chronic obstructive Lung Disease (GOLD) combined risk assessment score.[3]

## Statistics

**Figure 1.**
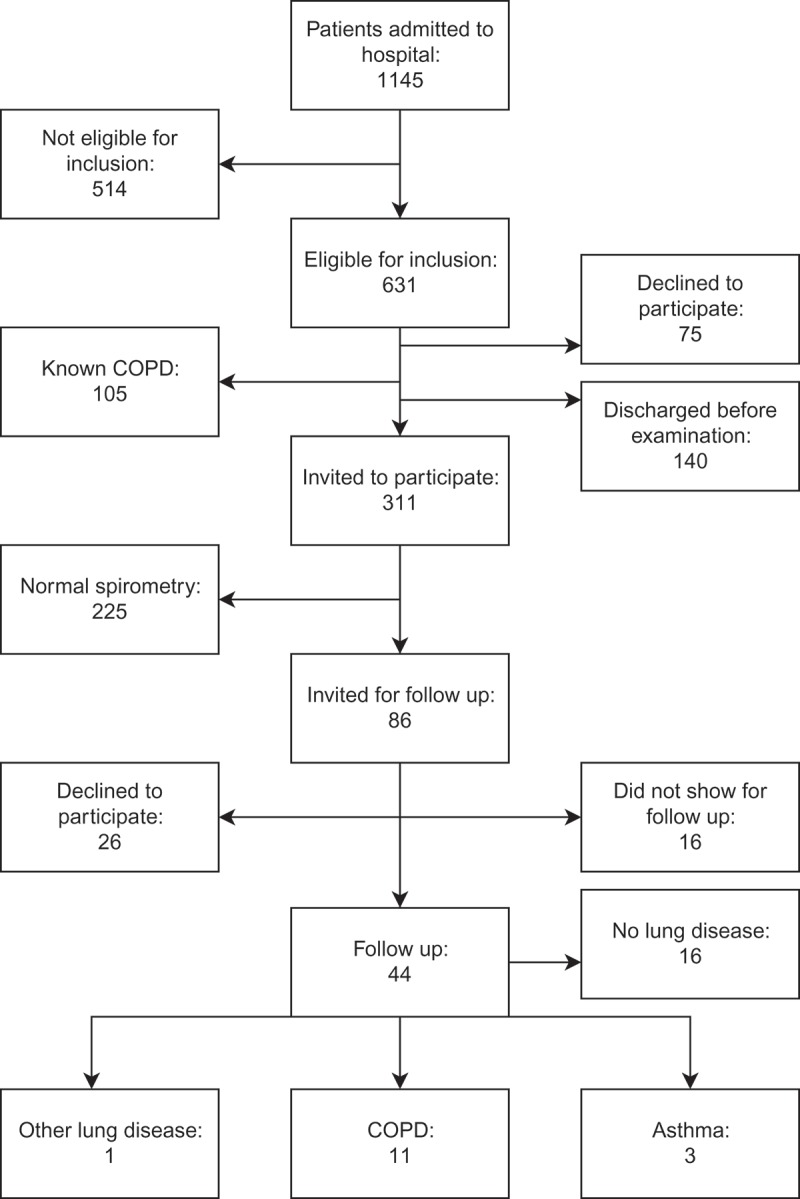
Flow chart of inclusion in the study.

Description of patient inclusion (Figure 1) was illustrated using Draw.io (Jgraph®, London, UK). All statistical work was done using SPSS® (IBM®, New York, NY, USA). Data were presented in median (quartiles), as they were not normally distributed, or %. Paired *t*-tests were performed comparing the registered data mentioned above for those with abnormal spirometry with patients with a normal spirometry. Furthermore, paired *t*-tests were also carried out comparing subgroups of those with abnormal spirometry (patients with obstructive spirometry; patients who declined a follow up; patients that did not show for the follow up and those who participated in follow up) with patients with a normal spirometry. *P*-values < 0.05 were considered significant.

Ethics: This study was approved by the Ethical Committee in the Northern Region of Denmark (N-20150005). All patients were informed according to the Helsinki Declaration and gave written consent prior to inclusion in the study.

## Results

### Patient inclusion

Figure 1 demonstrates the selection of patients for the study population and the investigational process during the study period. A total of 55%(631/1145) were considered eligible in terms of age, ability to understand information as well as to give informed consent and ability to perform spirometry; 46% (526/1145) were eligible when also excluding patients with known COPD, which in all three AAUs were 10% of the admitted patients. Of those, 28% (86/311) performed an abnormal spirometry at the initial examination.

### Patients’ characteristics

Table 1 shows the baseline characteristics of the entire study population; patients with a normal as well as abnormal spirometry at the primary investigation and subgroups of patients with abnormal spirometry at the primary examination, including those who participated at follow up. A total of 12% (36/311) of those investigated had obstructive lung function (FEV1/FEV6 < 0.7) at the primary examination.

**Table 1.** Basic caracteristics of the total study population expressed in median (qartiles) or %. Total numbers in [brackets].

	Total study population [311]	Normal lung function [225]	Referred to follow up [86]	Referred, FV1/FEV6 < 0.7 [36]	Declined follow up [26]	No show [16]	Follow up [44]
Age (years); median (quartiles)	59	54	70^1^	76^1^	74^2^	63^5^	70^3^
	(43–74)	(37–54)	(55–82)	(63–83)	(57–83)	(54–77)	(55–80)
Gender (male) (%)	48	42	60^3^	68^2^	52	63^3^	64^3^
Smoking status (never/former/present) (%)	41/30/28	47/26/26	26/40/33	22/42/36	24/48/28	25/38/38	29/29/35
Pack years;	3	1	13^4^	18^4^	8	10	23^3^
median (quartiles)	(0–20)	(0–1)	(0–34)	(2–34)	(1–34)	(1–24)	(0–38)
BMI (kg m^–^^2^);	25	25	26	24	26	26	26
median (quartiles)	(22–30)	(23–25)	(22–30)	(22–27)	(22–28)	(23–29)	(24–31)
Former lung function (%)	34	32	40	42	51	31	32

^1^ *p* < 0.0001 ^2^ *p* = 0.01 ^3^ *p* = 0.02 ^4^ *p* = 0.03 ^5^ *p* = 0.04 (*p*-values express significant differences between patients with normal lung function and the subgroup in question).

### Findings at primary examination

Table 2 shows the findings at the primary examination of the patients in the acute assessment units. Patients with abnormal lung function were significantly more symptomatic, judged by the MRC score and CAT, than those with normal lung function. Those who declined follow up were less symptomatic than those who accepted the offer. Patients with obstructive lung function did not have a significantly different CAT to those with normal lung function.

**Table 2.** MRC-score, CAT-score, FEV1 (total numbers and %), FEV6 (total numbers and %) and FEV1/FEV6 expressed in medians (Quartiles) in the total study population and subgroups. Total numbers in [brackets].

	Total study population [311]	Normal lung function [225]	Referred to follow up [86]	Referred, FEV1/FEV6 <0.7 [36]	Declined follow up [26]	No show [16]	Follow up [44]
MRC score;	1	1	2^2^	2^2^	1	2^4^	2^3^
median (quartiles)	(1–2)	(1–1)	(1–2)	(2–2)	(1–2)	(2–2)	(1–3)
CAT-score;	8	7	9^5^	8	7	10^3^	11^1^
median (quartiles)	(4–12)	(3–7)	(6–14)	(5–11)	(4–9)	(8–16)	(7–17)
FEV1 (liters);	2.6	2.8	1.7^1^	1.5^1^	1.4^1^	1.8^1^	1.9^1^
median (quartiles)	(1.7–3.0)	(2.3–2,8)	(1.2–2.2)	(1.0–2.0)	(1.0–1.9)	(1.3–2.5)	(1.2–2.3)
FEV1%; median (quartiles)	85	93	67^1^	66^1^	64^1^	68^1^	70^1^
	(70–98)	(84–93)	(51–72)	(48–72)	(51–70)	(50–73)	(65–72)
FEV6 (liters); median (quartiles)	3.2	3.5	2.4^1^	2.4^1^	2.2^1^	2.2^2^	2.9^1^
	(3.2–4.0)	(2.9–3.5)	(1.8–3.0)	(1.9–3.2)	(1.7–2.7)	(2.0–3.1)	(2.2–3.1)
FEV6%; median (quartiles)	91	97	70	82	72^1^	62^1^	74^1^
	(76–103)	(86–97)	(59–83)	(66–93)	(60–81)	(55–79)	(64–85)
FEV1/FEV6; median (quartiles)	0.80	0.81	0.69^1^	0.62^1^	0.69^1^	0.74	0.72^2^
	(0.73–0.85)	(0.77–0.81)	(0.62–0.81)	(0.56–0.66)	(0.62–0.81)	(0.64–0.84)	(0.60–0.82)

^1^ *p*<0.0001 ^2^ *p* = 0.01 ^3^ *p* = 0.02 ^4^ *p* = 0.03 ^5^ *p* = 0.04 (*p*-values express significant differences between patients with normal lung function and the subgroup in question).

### Patients referred to follow up

Of those offered a follow up in the outpatient clinic, 30% (26/86) declined further examination. Compared to those who participated in the follow up these patients were comparable in age, gender, BMI, smoking status and pack years. However, they had a significant better CAT score (*p* = 0.02) but did not differ in MRC (*p* = 0.2). They had a near significant better FEV1 (*p* = 0.06) and a significantly better FEV6 (*p* = 0.03), but did not differ in FEV1% (*p* = 0.4) or FEV6% (*p* = 0.6).

Of those who accepted a follow up 27% (16/60) did not show for the follow up. These patients did not differ from those who participated in the follow up in either basic characteristics or lung function.

### Findings at follow up

Of those referred to follow up 51% (44/86) accepted and showed for the follow up and 17% (15/86) were diagnosed with lung disease, 13% (11/86) diagnosed with COPD. Previously 11% (5/44) of those had been admitted to hospital with pulmonary infections, and 80% (4/5) of those were diagnosed with lung disease at the follow up.

From initial examination to follow up there was a median 10% increase in FEV1 (*p* = 0.01) and a median 12% increase in FEV6/FVC (*p* = 0.01). There were no significant changes in the ratio FEV1/FEV6 to FEV1/FVC.

Of those invited for follow up 42% (36/86) had FEV1/FEV6 < 0.7. Of those 33% (12/36) accepted and 75% (9/12) remained obstructive and 8% (1/12) were diagnosed with asthma.

Patients diagnosed with COPD had a median FEV1% of 70 (59-80) and a median FEV1/FVC 0.62 (0.56–0.67). Of those 18% (2/11) were GOLD spirometric stage 1, 45% (5/11) stage 2 and 27% (3/11) stage 3. All patients were either present (3/11) or former (8/11) smokers. Patients were distributed with 64% (7/11) in GOLD group A, 18% (2/11) in GOLD group B, and 9% (1/11) of both GOLD group C and D.

Asthma was diagnosed 7% (3/44) of the patients at follow up. Patients were diagnosed with a post bronchodilator increase in lung function of > 15% (2/3) and bronchial provocation with mannitol (1/3). Patients’ age ranged from 52 to 58 years, they were all never smokers and all had a MRC score of 1 and a CAT-score ranging from 7 to 12. Rhinitis was found in 2/3 of the patients. FEV1% ranged between 78 and 83% and FEV1/FVC between 0.68 and 0.74.

## Discussion

This study showed that about 50% of the patients admitted to the AAU were able to perform a spirometry and about 25% of those had an abnormal spirometry at the initial examination. Half of the patients offered a follow up accepted and showed, and nearly 17% of these were diagnosed with lung disease, 13% with COPD. Three-quarter of the patients who had obstructive lung function at the initial examination remained obstructive and in addition one of these patients was diagnosed with asthma. Patients diagnosed with COPD primarily classified as GOLD group A.

Previously, only a limited number of COPD screening studies have been carried out in hospitalized populations. These studies have found between 25 and 34% of the study populations to have undiagnosed COPD.[9,16] This is not consistent with the findings of this study where a considerably smaller proportion the examined patients were diagnosed with COPD. There are several possible explanations of this. In the study by Kart et al. [16] examining outpatients and patients hospitalized for elective treatment, a considerably larger proportion of the study population were able to participate in spirometry; in the study by Nielsen et al. [9] all inpatients were examined, and in neither of these studies the study populations were followed up in stable state. In fact, in this study a larger proportion of the study population had airflow obstruction at the initial examination than in the above-mentioned studies. As only half of those with initial abnormal lung function were examined at follow up one might speculate that the proportion of patients with COPD in our study population is in reality higher than what was diagnosed at the follow up. This does suggest that patients in need of acute hospital admission may be a relevant population to investigate with spirometry in future.

In this study patients with air flow obstruction at the initial examination were significantly more symptomatic, judged by both MRC and CAT score. This is even though those diagnosed with COPD only had mild COPD. This is consistent with previous studies, which also showed newly diagnosed, mild COPD patients to be symptomatic.[17–19] In addition, more symptomatic patients with airflow obstruction were older and had a smoking history. This is also consistent with previous studies [20–22] and the Global Initiative for Chronic Obstructive Lung Disease, GOLD, also recommends to perform spirometry in patients with a smoking history and respiratory symptoms.[3] As such, this study supports this recommendation and demonstrates that this is also the case in hospitalized patients.

Asthma was diagnosed in 7% of the patients at follow up. This is exactly the same number as was found in the Copenhagen City Heart Study.[23] The lung function in these patients is also similar to that found in the study by Porsbjerg et al.,[23] i.e. about 80%. Rhinitis was highly prevalent in the asthma patients, and the fact that these to diagnoses co-exist is also well known.[24]

Almost half of the patients referred to follow up did not accept the referral or did not show, which of course is a major limitation of this study. It does however also point to a very important and difficult question in achieving early diagnosis of COPD: patients’ reluctance to get the diagnosis. Suggested explanations have been that patients harbor guilt for their smoking history or are reluctant to quit smoking;[25] in this study population a larger, although not significantly larger, proportion of those who participated in the follow up were active smokers compared to those who did not participate. A lack of awareness of symptoms have also been proposed as a patient barrier;[25] in this study patients were able to identify symptoms. A previous study has indicated that lack of knowledge about COPD influence patients’ self-care; however, this has not been investigated in this study.[26] A sense of therapeutic nihilism in the clinicians transmitted to patients has also been proposed, but has this not been addressed in this study. Regardless, this is a question that needs further investigation in the future.

Only half of the patients admitted to the AAU were eligible to participate in this study. This is of course also a limitation to this study; this may lead to underestimation of patients with abnormal lung function. As COPD patients often suffer from comorbidities, leading to increased morbidity, it is possible that COPD may be frequent in those too ill to participate.[27] On the other hand, a number of patients admitted to the AAU were too young to meet the age criteria for inclusion, which may lead to an overestimation of abnormal lung function in patients in need of acute admission. It is of course questionable whether the AAU is an appropriate site for screening to COPD. Possibly the patients should not be examined within the first 24 h of admission, but, for example, when preparing hospital discharge. If focusing on COPD, all patients diagnosed with COPD had a smoking history. Therefore, if, in future, spirometry was offered to patients with symptoms and a smoking history, and follow up was offered to those with obstructive lung function, this study has proved that this is a feasible, and efficient way to identify undiagnosed COPD patients. It is also noteworthy that even though lung function improved by 10% from the initial spirometry to follow up, three-quarter of the patients remained obstructive.

In conclusion, approximately half of the patients in need of acute hospitalization were able to perform spirometry and 12% of these patients had airflow obstruction at initial examination. The AAU may as such be a possible site of screening for COPD in future.
